# Disrupted Regional Cerebral Blood Flow and Functional Connectivity in Pontine Infarction: A Longitudinal MRI Study

**DOI:** 10.3389/fnagi.2020.577899

**Published:** 2020-11-19

**Authors:** Ying Wei, Luobing Wu, Yingying Wang, Jingchun Liu, Peifang Miao, Kaiyu Wang, Caihong Wang, Jingliang Cheng

**Affiliations:** ^1^First Affiliated Hospital of Zhengzhou University, Zhengzhou, China; ^2^Department of Radiology and Tianjin Key Laboratory of Functional Imaging, Tianjin Medical University General Hospital, Tianjin, China; ^3^GE Healthcare MR Research, Beijing, China

**Keywords:** pontine infarction, cerebral blood flow, functional connectivity, motor, cognition

## Abstract

Abnormal cerebral blood flow (CBF) and resting-state functional connectivity (rs-FC) are sensitive biomarkers of disease progression and prognosis. This study investigated neural underpinnings of motor and cognitive recovery by longitudinally studying the changes of CBF and FC in pontine infarction (PI). Twenty patients underwent three-dimensional pseudo-continuous arterial spin labeling (3D-pcASL), resting-state functional magnetic resonance imaging (rs-fMRI) scans, and behavioral assessments at 1 week, 1, 3, and 6 months after stroke. Twenty normal control (NC) subjects underwent the same examination once. First, we investigated CBF changes in the acute stage, and longitudinal changes from 1 week to 6 months after PI. Brain regions with longitudinal CBF changes were then used as seeds to investigate longitudinal FC alterations during the follow-up period. Compared with NC, patients in the left PI (LPI) and right PI (RPI) groups showed significant CBF alterations in the bilateral cerebellum and some supratentorial brain regions at the baseline stage. Longitudinal analysis revealed that altered CBF values in the right supramarginal (SMG_R) for the LPI group, while the RPI group showed significantly dynamic changes of CBF in the left calcarine sulcus (CAL_L), middle occipital gyrus (MOG_L), and right supplementary motor area (SMA_R). Using the SMG_R as the seed in the LPI group, FC changes were found in the MOG_L, middle temporal gyrus (MTG_L), and prefrontal lobe (IFG_L). Correlation analysis showed that longitudinal CBF changes in the SMG_R and FC values between the SMG_R and MOG_L were associated with motor and memory scores in the LPI group, and longitudinal CBF changes in the CAL_L and SMA_R were related to memory and motor recovery in the RPI group. These longitudinal CBF and accompany FC alterations may provide insights into the neural mechanism underlying functional recovery after PI, including that of motor and cognitive functions.

## Introduction

Pontine infarction (PI) accounts for 7% of ischemic strokes and is the most common type of posterior circulation infarction (Wang et al., 2018). Typical clinical manifestations of PI include pure motor hemiplegia, ataxia, dysarthria, dysphagia, and cognitive impairment (Kinjo et al., 2017; Lapa et al., 2017; Li et al., 2020). Long-term follow-up has revealed that some patients with PI experience a poor prognosis with impaired motor or cognitive function (Mijajlovic et al., 2017; Payabvash et al., 2018). Therefore, understanding the neural mechanisms of behavioral impairment and recovery is crucial in stroke rehabilitation.

In the past few years, neuroimaging studies have been widely used to explore neurobiological mechanisms in the brain under healthy and pathological conditions and have been shown to be useful to predict further behavioral recovery (Haque et al., 2019; Xie et al., 2019; Zhang et al., 2020). Recently, an increasing number of studies have reported that abnormal cerebral hemodynamics represent an important pathophysiological mechanism in the post-stroke brain and that cerebral blood flow (CBF) is disturbed not only in the infarction or peri-infarct areas but also in the remote brain regions that are correlated with injury brain areas (Miao et al., 2018; Wang et al., 2019). Previous studies using single-photon emission computed tomography (SPECT) have also suggested that abnormal perfusion in remote areas, including the cerebellum and supratentorial frontoparietal cortex after PI, and disruption and reorganization of CBF are two of the mechanisms underlying behavioral impairment and improvement (Hoffmann and Watts, 1998; D’Aes and Marien, 2015). However, issues with exposure to ionizing radiation and the use of exogenous tracer agents have limited clinical researches, particularly studies requiring repetitive imaging and longitudinal researches.

Arterial spin labeling (ASL) imaging can quantitatively evaluate regional CBF values using arterial blood as an endogenous diffusion tracer (Deibler et al., 2008). Currently, ASL is a promising alternative technique for investigating the neural basis of various neurological and psychiatric diseases, such as stroke, seizures, Alzheimer’s disease, and Parkinson’s disease, since the perfusion results detected by ASL are consistent with those detected by conventional techniques. Additionally, ASL detection is non-invasive and non-ionizing (Le Heron et al., 2014; Wiest et al., 2014; Xie et al., 2019; Zhang et al., 2020). Some researchers have investigated CBF patterns of chronic PI by using ASL and have found that patients with PI showed higher CBF values in the contralateral inferior frontal gyrus and lower CBF values in the bilateral cerebellum; moreover, the CBF values in the contralateral cerebellum were closely associated with motor function scores (Wang et al., 2019). However, the processes underlying CBF changes are intrinsic and dynamic during the period of functional damage and subsequent recovery; thus, a longitudinal study is important for gaining comprehensive understanding of the neurobiological underpinnings.

Neurological activity in the brain regions is accompanied by changes of CBF and blood oxygenation, which can be measured by functional magnetic resonance imaging (fMRI) techniques (Detre and Wang, 2002; Fox and Raichle, 2007; Boscolo Galazzo et al., 2014). Resting-state functional connectivity (FC), which is based on detection of the temporal correlations of blood oxygen level-dependent (BOLD) signals between two brain regions, has been used to investigate the cortical reorganization and compensation (Buckner et al., 2013; Guerra-Carrillo et al., 2014). Although early evidence has utilized FC to investigate the neural mechanisms underlying stroke-related behavioral function decline, there is little knowledge about PI, especially longitudinal and within-person changes. In cross-sectional studies, patients with acute PI showed significantly lower FC in the medial prefrontal cortex and precuneus (Jiang et al., 2018), while patients with early chronic PI exhibited decreased FC in the medial prefrontal gyrus, precuneus, right posterior cingulum, and left middle cingulum gyrus (Chen et al., 2019). In a longitudinal-sectional study, some brain regions exhibited special changes during the follow-up period, in which voxel-mirrored homotopic connectivity (VMHC) in the precentral and postcentral gyrus tended to increase gradually, and VMHC in the hippocampus/amygdala was increased throughout the study period, and VMHC in the hippocampus/amygdala and frontal pole in the early stages were associated with the later motor recovery (Shan et al., 2018).

In addition, recent studies have confirmed the association between CBF and FC, and FC might be modulated by regional CBF of the seed (Li et al., 2012; Liu et al., 2013). Network analysis has found that specific network nodes present a higher CBF to main proper network integrity (Liang et al., 2013). According to the neurovascular coupling hypothesis, altered CBF may be coupled with significant changes in the brain FC (Venkat et al., 2016; Zhu et al., 2017). Therefore, some researches have attempted to explore pathophysiological mechanisms and effectiveness of drug therapy in neuropsychiatric disorders using multimodal MRI such as ASL and BOLD fMRI (He et al., 2019; Tan et al., 2020).

Based on the abovementioned knowledge, in order to explore the neural substrates of functional recovery after PI, we first investigated the cross-sectional and longitudinal changes in CBF during the 6 months following PI. After identifying the longitudinal changes in CBF, the progressive FC changes were further explored using these CBF-altered regions as seed. Finally, we also further investigated the relationships among abnormal changes in CBF, FC, and behavioral performance after stroke.

## Materials and Methods

### Participants

Participants were recruited from the First Affiliated Hospital of Zhengzhou University and Tianjin Medial University General Hospital. These subjects provided written informed consent before the study, and the study protocol was approved by the local Research Ethics committee.

The inclusion criteria were as follows: (1) first-onset infarction and within 1 week after onset of symptoms; (2) right-handedness before brain stroke; (2) age from 40 to 80 years; (3) unilateral lesion located in the pontine identified, as identified by MRI examination; and (4) adherence to follow-up tests for 6 months. The exclusion criteria were as follows: (1) secondary hemorrhage and recurrent stroke during follow-up; (2) the score of artery stenosis >2 based on magnetic resonance angiography (Ferguson et al., 1999); (3) severe white matter hyperintensity with a Fazekas scale score >1; (4) history of drug dependence or concomitant neuropsychiatric disorders; (5) severe systemic comorbidities, including heart failure and cancer; and (6) contraindications for MRI. Finally, 20 patients with unilateral PI were recruited from October 2014 to May 2019, including 10 subjects with lesions on each side. Twenty age-, sex-, and education-matched normal control (NC) subjects were recruited as a control group.

The infarction locations at the acute phase were manually delineated based on the acute diffusion-weighted imaging by using MRIcron software^1^. The lesion masks of all stroke patients were normalized in the Montreal Neurological Institute (MNI) space and then overlaid on a normalized template to generate the lesion distribution probability maps.

### Image Data Acquisition

MRI examinations were performed using a 3.0-Tesla MR imaging system (Discovery MR 750, GE Medical Systems, Waukesha, WI, United States) at 1 week, 1, 3, and 6 months after infarction onset in the PI group, while the NC subjects were examined only once in the same manner. All subjects were instructed to hold still, close their eyes, and think of nothing in particular. Comfortable foam padding and earplugs were used to reduce head motion and scanner noise. Brain perfusion imaging was performed using a three-dimensional pseudo-continuous arterial spin labeling (3D-pcASL) sequence with the following parameters: TR/TE = 5,025 ms/11.1 ms; post label delay = 2,025 ms; spiral in readout of eight arms with 512 sample points; FA = 111°; field-of view (FOV) = 240 × 240 mm; matrix = 128 × 128; slice thickness = 3.0 mm, no gap, 48 slices, number of excitations = 3, and 1.9 × 1.9 mm in-plane resolution. Spatial-3D T1-weighted images were acquired by using a brain volume sequence with the following imaging parameters: TR/TE = 8.2 ms/3.2 ms; FA = 12°; FOV = 256 × 256 mm; matrix = 256 × 256; slice thickness = 1.0 mm; no gap; 188 slices. Resting-state fMRI data were acquired by using a gradient-echo single-shot echo-planar imaging sequence with the following imaging parameters: TR/TE = 2,000 ms/41 ms; FA = 90°; FOV = 220 × 220 mm; matrix = 64 × 64; slice thickness = 4 mm; 0.5 mm gap; 32 slices; 190 time points. Diffusion-weighted imaging sequences were acquired at the axial plane with TR/TE = 6,000 ms/96 ms; FOV = 256 × 256 mm; slice thickness = 5.0 mm; 1.5 mm gap; 21 slices.

### Behavioral Evaluation

Clinical stroke severity was assessed using the National Institutes of Health Stroke Scale (NIHSS), which is a 15-item stroke scale and score ranging from 0 to 42, with a higher score indicating a more serious function impairment (Kasner, 2006). They also underwent more detailed behavioral examination about motor and memory functions. Motor impairment was assessed using the Fugl–Meyer test (FMT) for upper and lower extremities, which is a standardized and reliable motor impairment scale. The score ranges from 0 to 100, with the higher score indicating better performance. The Ray Auditory Verbal Learning Test (RAVLT) was used to explore short-term verbal memory function, and the number of correctly recalled words was recorded as the RAVLT scores.

### Image Processing

The CBF images were derived from the pcASL difference images by subtracting the labeled images from the control images. CBF images were processed using the SPM8 toolbox^2^. First, the individual 3D T1 images were coregistered to the individual ASL images to produce the new individual 3D T1 images. Second, the new individual 3D T1 images were normalized to a standard T1 template in MNI space and segmented into gray matter (GM), white matter, and cerebrospinal fluid maps. Third, the individual CBF images were subsequently written to the spatially normalized 3D T1 GM maps in MNI space and the voxel size for the normalized written images was 2 × 2 × 2 mm^3^. Fourth, to reduce individual variance, the spatially normalized CBF maps were transformed into *z* scores by subtracting the mean and dividing by the standard deviation (SD) of the global value within the GM mask. Finally, the images were spatially smoothed using a Gaussian kernel of 6-mm full width at half-maximum (FWHM).

Resting-state functional MRI (rs-fMRI) data were preprocessed using Data Processing and Analysis of Brain Imaging^3^ (DPABI) in the SPM 8 software package. The preprocessing included the following steps. First, the first 20 volumes of subjects were removed to allow the signal to reach equilibrium. Second, the remaining 170 volumes were corrected for time delay between slices. For the head motion, we used a threshold of 2 mm translation and 2° rotation, and no subject exceeded this maximum. In this step, the mean framewise displacement (mean FD) was also calculated to characterize the mean head motion (Power et al., 2012). Third, functional images were co-registered to the 3D T1 images, and the remaining dataset was spatially normalized to MNI space and resampled into a voxel size of 3 × 3 × 3 mm^3^. Fourth, the images were smoothed by using a Gaussian kernel of 6-mm FWHM, and several covariates were regressed via linear regression. Finally, a temporal band-pass frequency filter (0.01–0.08 Hz) was performed to reduce low-frequency drift and high-frequency noise. We used the seed-based functional connective method to explore the different FC values over time in PI patients. Brain regions with significant longitudinal CBF changes in the left pontine infarction (LPI) and right pontine infarction (RPI) groups were respectively defined as seed regions of interest (ROIs), and Pearson’s correlation coefficients were calculated between the mean time series of each ROI and each voxel in the whole brain GM as the seed-based FC maps. Then, the FC maps were transformed into Fisher’s *z* maps for group analysis.

### Statistic Analysis

The data at different time points in the same patients were divided into four subgroups, depending on the follow-up time. The demographic differences between the LPI, RPI, and NC groups were analyzed using independent two-sample *t*-tests or chi-square tests in the SPSS 21.0 software (SPSS, Inc., Chicago, IL, United States). The normality of clinical data distribution was tested using the Shapiro–Wilk test. A two-sample *t*-test was used to analyze behavioral differences between baseline subgroups of the PI and NC groups. One-way repeated-measure analysis of variance (ANOVA) and Bonferroni’s *post hoc* tests were used to investigate the mean FD and behavioral changes between different LPI and RPI subgroups. All tests were two-tailed, and the significance threshold was set at *P* < 0.05.

For the cross-sectional comparison of CBF at the baseline stage between PI and NC groups, a voxel-wise two-sample *t*-test was performed, with age, sex, and education years as the covariance factors. For longitudinal comparisons, one-way repeated-measure ANOVA and Bonferroni’s *post hoc* tests were used to explore the significance of differences in CBF and secondary FC changes among various time-related subgroups in the LPI and RPI groups. In addition, multiple comparisons were corrected using the AlphaSim program (the voxel-wise, *P* < 0.001; the cluster-wise, *P* < 0.005; 5,000 simulations) in the REST toolbox^4^ 1.8. Pearson’s or Spearman’s correlation coefficients were calculated to test the association of normalized *z*-scored CBF values of each brain region with longitudinal CBF changes and behavioral scores over time. The association between the normalized *z*-scored FC values of each brain region showing longitudinal FC changes and motor and memory scores was determined using the same methods.

## Results

### Demographic and Behavioral Measures

**FIGURE 1:**
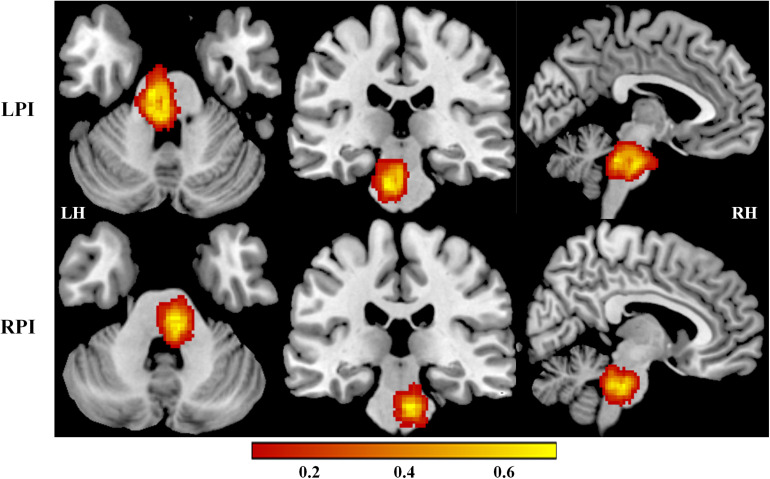
Lesion distribution maps in the LPI and RPI groups. The color bar represents the lesion probability. LH, left hemisphere; RH, right hemisphere; LPI, left pontine infarction; RPI, right pontine infarction.

Demographic and clinical characteristics of LPI, RPI, and NC subjects are shown in Table 1, Supplementary Figure S1, and Supplementary Table S1 in the Supplementary Material. Compared with NC subjects, there were no significant differences in age (*P*′_LPI_ = 0.06, *P*′_RPI_ = 0.64), gender (*P*′_LPI_ = 0.64, *P*′_RPI_ = 0.28), education years (*P*′_LPI_ = 0.45, *P*′_RPI_ = 0.13), and the incidence of other concurrent disease in the LPI and RPI groups. In addition, no significant differences in lesion volume (*P* = 0.11) and the days from stroke to the first follow-up (*P* = 0.71) were observed between the LPI and RPI groups in the baseline stage. Compared with the NC group, the LPI and RPI groups in the baseline stage showed significant motor and verbal memory impairment (RAVLT scores, *P*′_LPI_ < 0.001, *P*′_RPI_ = 0.01). Longitudinal clinical examination in the LPI and RPI patients showed significant improvement over time in FMT (*P*_LPI_ = 0.001, *P*_RPI_ = 0.005) and RAVLT scores (*P*_LPI_ < 0.0001, *P*_RPI_ < 0.0001), but only the LPI group showed significant decrease in NHISS scores (*P*_LPI_ = 0.002, *P*_RPI_ = 0.07). Furthermore, there was no significant difference among different subgroups in mean FD values (*P*_LPI_ = 0.349, *P*_RPI_ = 0.864). The lesion distribution probability maps of the LPI and RPI groups in the acute stage are depicted in Figure 1.

**TABLE 1:** Demographic and clinical data in the PI and NC groups.

Variable	PI (*n* = 20)		NC (*n* = 20)	*P*-value
	LPI (*n* = 10)	RPI (*n* = 10)		
Age, years	60.10 ± 4.40	54.40 ± 7.87	55.67 ± 6.24	P′_LPI_ = 0.06; P′_RPI_ = 0.64
Gender (male/female)	7/3	4/6	12/8	P′_LPI_ = 0.64; P′_RPI_ = 0.28
Years of formal education	10.20 ± 3.52	9.60 ± 1.89	11.11 ± 2.72	P′_LPI_ = 0.45; P′_RPI_ = 0.13
Lesion size at acute phase(ml)	2.75 ± 1.86	1.66 ± 0.75	/	*P* = 0.11
Stroke to the first follow-up (days)	4.90 ± 1.85	4.60 ± 1.71	**/**	*P* = 0.71
**Medical history**				
Coronary heart disease	1/10	1/10	0/20	P′_LPI_ = 0.39; P′_RPI_ = 0.39
Hypertension	4/10	5/10	4/20	P′_LPI_ = 0.23; P′_RPI_ = 0.09
Hyperlipidemia	4/10	2/10	2/20	P${}_{\text{LPI}}^{\prime }$ = 0.05; P′_RPI_ = 0.45
Diabetes	1/10	2/10	1/20	P′_LPI_ = 0.61; P′_RPI_ = 0.19
Smoking	2/10	3/10	2/20	P′_LPI_ = 0.45; P′_RPI_ = 0.17
Drinking	1/10	1/10	1/20	P′_LPI_ = 0.61; P′_RPI_ = 0.61
**NIHSS score**				
Time 1	3.50 ± 3.92	1.50 ± 2.50		/;/
Time 2	1.70 ± 2.35	1.20 ± 2.09	**/**	F = 9.57; P_LPI_ = 0.002*
Time 3	1.00 ± 2.21	0.20 ± 0.63		*F* = 2.65; P_RPI_ = 0.07
Time 4	0.50 ± 1.26	0.10 ± 0.31		
**FMT**				
Time 1	73.90 ± 33.48	83.20 ± 22.95		/;/
Time 2	85.90 ± 26.52	96.30 ± 7.48	**/**	*F* = 7.19; P_LPI_ = 0.001*
Time 3	89.60 ± 25.83	98.40 ± 5.06	**/**	*F* = 5.40; P_RPI_ = 0.005*
Time 4	91.20 ± 21.87	99.00 ± 3.16		
**RAVLT**				
Time 1	34.00 ± 7.11	37.60 ± 11.27	48.11 ± 8.44	P′_LPI_ < 0.001*; P′_RPI_ = 0.01*
Time 2	44.80 ± 7.96	49.50 ± 10.59		*F* = 18.21; P_LPI_ < 0.001*
Time 3	50.40 ± 12.44	53.00 ± 11.19		*F* = 13.17; P_RPI_ < 0.001*
Time 4	52.20 ± 7.94	58.88 ± 10.56		
**Mean FD**				
Time 1	0.116 ± 0.042	0.138 ± 0.047	/	*F* = 1.158; P_LPI_ = 0.349
Time 2	0.144 ± 0.042	0.146 ± 0.082		*F* = 0.245; P_RPI_ = 0.864
Time 3	0.139 ± 0.036	0.121 ± 0.073		
Time 4	0.131 ± 0.038	0.134 ± 0.046		

*Data were presented as mean ± SD for continuous data. P_PI_′, P values of demographic data and behavioral test scores between the baseline stage of PI and NC groups; P_PI_, P value of behavioral scores among PI subgroups at different time-points; P, P value of infarct volume at acute phase between LPI and RPI groups. Time 1, Time 2, Time 3, and Time 4, different time subgroups from 1 week to 6 months after PI. LPI, left pontine infarction; RPI, right pontine infarction; NC, normal control; NIHSS, National Institutes of Health Stroke Scale; FMT, Fugl–Meyer Test; RAVLT, Ray Auditory Verbal Learning Test; Mean FD, mean framewise displacement (FD). ^∗^p < 0.05.*

### Comparison of CBF Between the Baseline Subgroup of the PI and NC Groups

**FIGURE 2:**
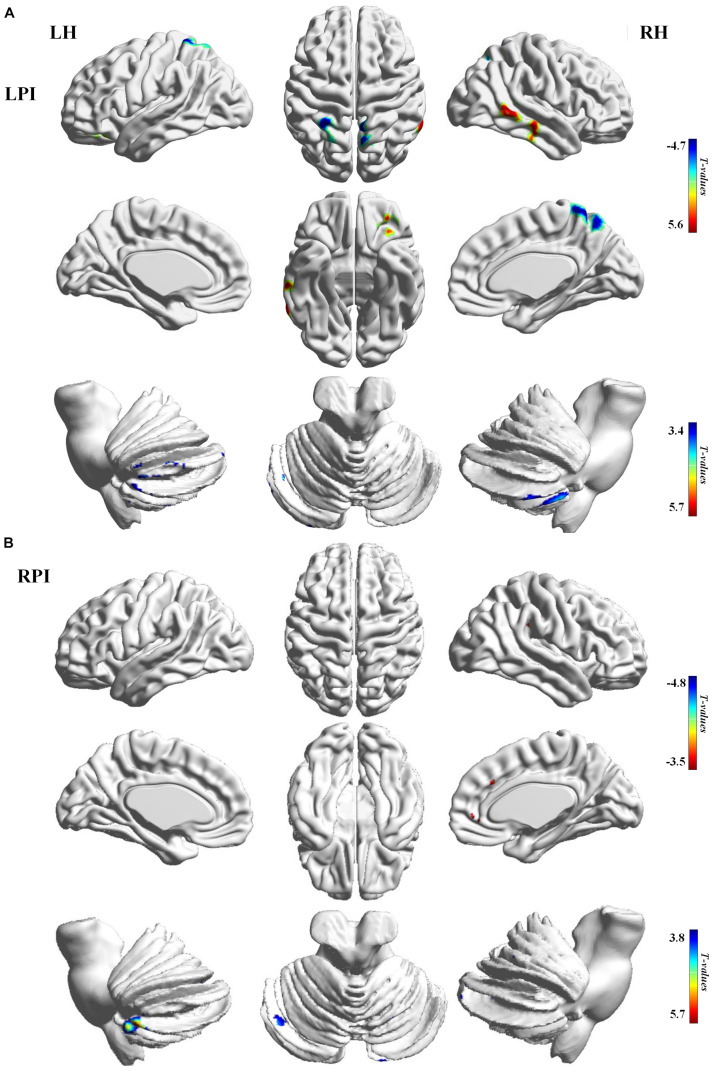
CBF differences between the baseline subgroups of the PI and NC groups. **(A)** Voxel-base analysis showed brain regions with significant CBF alterations in the baseline stage of the LPI group. **(B)** Voxel-base analysis showed brain regions with significant CBF alterations in the baseline stage of the RPI group. The color bar indicated the *t* scores. LH, left hemisphere; RH, right hemisphere; LPI, left pontine infarction; RPI, right pontine infarction.

Compared to the NC group, patients with LPI at the baseline stage showed lower *z*-scored CBF values in the bilateral cerebellum, including the left lobule VIII (CBE lobule VIII_L), Crus I (CBE lobule Crus I_L), right lobule VIIB (CBE lobule VIIB_R), the left inferior frontal gyrus (IFG_L), the left insula (INS_L), and right inferior and middle temporal gyrus (ITG_R, MTG_R). In addition, the brain regions with significantly higher *z*-scored CBF values were found in the right precuneus (PCUN_R) and left superior parietal gyrus (SPG_L) (AlphaSim correction, *P* < 0.005, cluster size = 43). Patients with RPI at baseline showed significantly lower *z*-scored CBF values in the bialteral cerebellum including left lobule VIII (CBE lobule VIII_L), right lobule VI (CBE lobule VI_R), and Crus II (CBE Crus II_R) and higher *z*-scored CBF values in the right superior frontal gyrus (SFG_R) and right precentral gyrus (PreCG_R) (AlphaSim correction, *P* < 0.005, cluster size = 43) (Table 2, Figure 2).

**TABLE 2:** Brain regions with different CBF between the baseline stage of PI and NC group.

Brain regions	Cluster size (voxels)	Peak intensity	MNI coordinates
***LPI group***			
CBE lobule VIII_L	293	5.550	(−20, −68, −60)
CBE lobule VIIB_R	181	5.691	(44, −64, −54)
CBE Crus I_L	109	3.959	(−52, −48, −38)
IFG_L	140	5.599	(62, −18, −28)
INS_L	77	4.744	(−30, 34, −20)
ITG_R	219	5.268	(−42, −16, 2)
MTG_R	176	5.481	(66, −42, 8)
PCUN_R	343	–4.736	(8, 50, 66)
SPG_L	301	–4.611	(−24, −58, 66)
***RPI group***			
CBE lobule VIII_L	355	5.773	(−38, −42, −46)
CBE lobule VI_R	440	5.698	(20, −60, −28)
CBE Crus II_R	87	5.009	(16, −88, −32)
SFG_R	79	–4.733	(22, 26, 64)
PreCG_R	87	–4.322	(20, −20, 80)

*CBE lobule VIII_L, left cerebellar lobule VIII; CBE Crus I_L, left cerebellar Crus I; CBE lobule VIIB_R, right cerebellar lobule VIIB; IFG_L, left inferior frontal gyrus; INS_L, left insula; ITG_R, right inferior temporal gyrus; MTG_R, right middle temporal gyrus; PCUN_R, right precuneus; SPG_L, left superior parietal gyrus; CBE lobule VI_R, right cerebellar lobule VI; CBE Crus II_R, right cerebellar Crus II; SFG_R, right superior frontal gyrus; PreCG_R, right precentral gyrus; LPI and RPI, left and right pontine infarction.*

### Longitudinal CBF Analysis During the Follow-Up Period After PI

**FIGURE 3:**
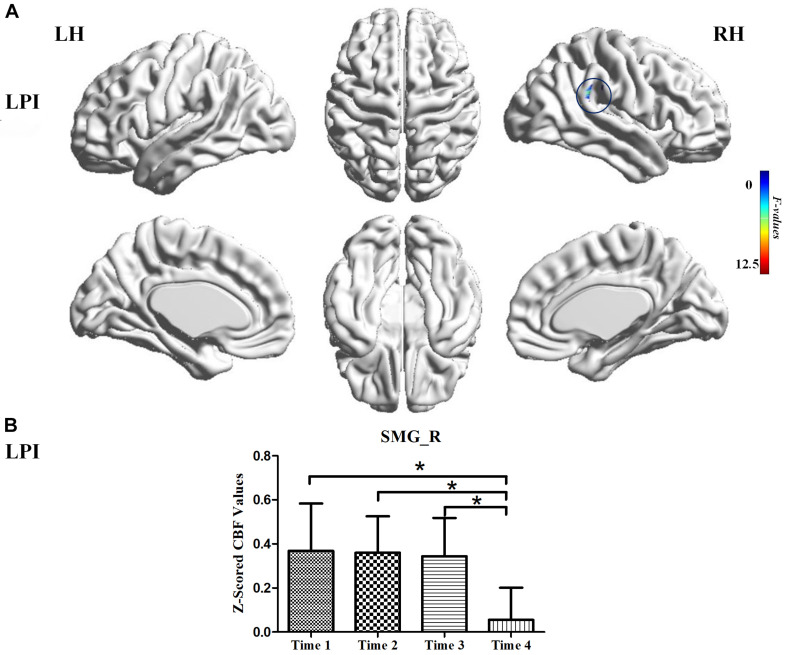
CBF differences among different time-subgroups in the LPI group. **(A)** One-way repeated-measure ANOVA showed brain regions with CBF differences among four follow-up subgroups in the SMG_R. The color bar indicated the *F* scores. **(B)** Bar plots showed the *z*-scored CBF values of the SMG_R at four different time-subgroups and the change trend from the baseline stage to 6 months after infarction. SMG_R, right supramarginal gyrus; Time 1, Time 2, Time 3, and Time 4, different time subgroups from 1 week to 6 months after infarction; LH, left hemisphere; RH, right hemisphere; LPI, left pontine infarction. *P < 0.05.

**FIGURE 4:**
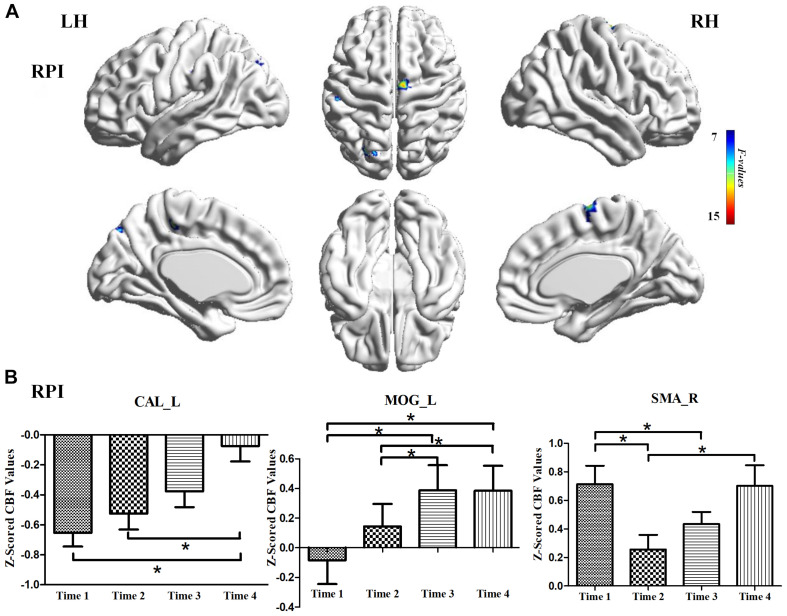
CBF differences among different time-subgroups in the RPI group. **(A)** One-way repeated-measure ANOVA showed brain regions with CBF differences among different time-point subgroups in the CAL_L, MOG_L, and SMA_R. color bar indicated the *F* scores. **(B)** Bar plots showed the *z*-scored CBF values of these significant brain regions at four different time-subgroups and the change trends from the baseline stage to 6 months. CAL_L, the left calcarine sulcus; MOG_L, left middle occipital gyrus; SMA_R, right supplementary motor area; LH, left hemisphere; RH, right hemisphere; RPI, right pontine infarction. *P < 0.05.

For the LPI group, among the four different time subgroups, patients only exhibited significant CBF changes in the right supramarginal gyrus (SMG_R) after the repeated-measure ANOVA test (AlphaSim correction, *P* < 0.005, cluster size = 54). Moreover, *post hoc* analyses revealed significantly lower *z*-scored CBF values of the SMG_R in the Time 4-subgroup (Bonferroni’s *post hoc* test, *P* < 0.001) (Table 3, Figure 3). For the RPI group, the patients showed longitudinal changes of CBF in the left calcarine sulcus (CAL_L), left middle occipital gyrus (MOG_L), and right supplementary motor area (SMA_R) (AlphaSim correction, *P* < 0.005, cluster size = 54) (Table 3, Figure 4). In addition, the *z*-scored CBF values in CAL_L and MOG_L displayed significantly increased trends during the follow-up period. In SMA_R, the *z*-scored CBF values are initially high in the baseline stage, decreasing to a minimum at 1 month, then increasing with time (Bonferroni’s *post hoc* test, all *P* < 0.001) (Figure 4).

**TABLE 3:** Brain regions with longitudinal CBF changes.

Brain regions	Cluster size (voxels)	Peak intensity	MNI coordinates
***LPI group***			
SMG_R	58	12.472	(66, −34, 28)
***RPI group***			
CAL_L	56	9.783	(−10, −102, −12)
MOG_L	127	14.131	(−24, −74, 32)
SMA_R	75	11.436	(8, −10, 68)

*SMG_R, right supramarginal; CAL_L, left calcarine sulcus; MOG_L, left middle occipital gyrus; SMA_R, right supplementary motor area; LPI, left pontine infarction; RPI, right pontine infarction.*

### Longitudinal FC Analysis in the LPI and RPI Groups

**FIGURE 5:**
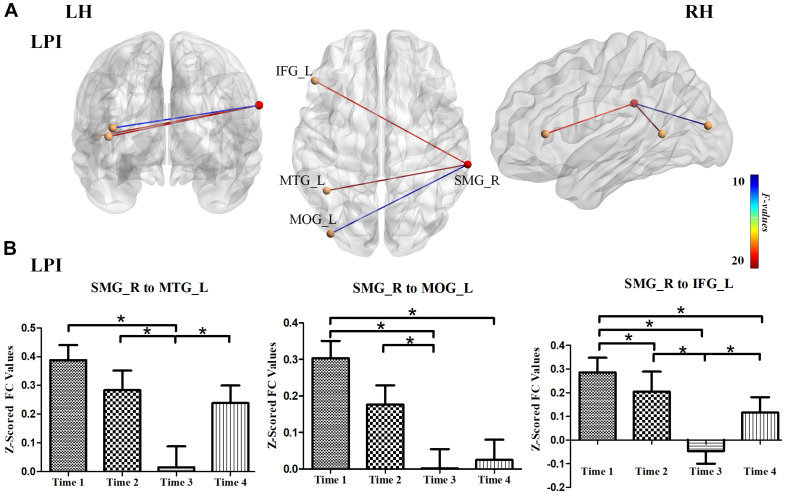
Longitudinal FC changes among different time-subgroups in the LPI group. **(A)** The results for longitudinal analysis of FC using the SMG_R as seed in the LPI group. The red ball represented the seed-ROI, and yellow balls represented brain regions with longitudinal FC changes with seed-ROI. The color of the line indicated *z*-scored FC values. **(B)** Bar plots showed the *z*-scored FC values in significant brain regions with longitudinal FC changes and the change trends over four time points. SMG_R, right supramarginal gyrus; MTG_L, left middle temporal gyrus; MOG_L, left middle occipital gyrus; IFG_L, left inferior frontal gyrus; LH, left hemisphere; RH, right hemisphere; LPI, left pontine infarction. *P < 0.05.

We further explored longitudinal FC alterations during the follow-up period in the LPI and RPI groups, using brain regions with altered CBF in longitudinal analysis as seed-ROIs. In the LPI group, with the SMG_R as the seed-ROI, patients exhibited significantly altered FC in the left middle temporal gyrus (MTG_L), left middle occipital gyrus (MOG_L), and left inferior frontal gyrus (IFG_L) (AlphaSim correction, *P* < 0.005, cluster size = 16) (Table 4, Figure 5). Interestingly, *z*-scored FC values in these brain regions showed similar trends of gradual decrease to a minimum at the 3-month time-point, and subsequent increase (Bonferroni’s *post hoc* test, all *P* < 0.001) (Figure 5), while in the RPI group, there were no brain regions with significant longitudinal differences among the four time-points.

**TABLE 4:** Brain regions with longitudinal FC changes.

Seed	Brain regions	Cluster size (voxels)	Peak intensity	MNI coordinates
SMG_R	MTG_L	54	19.297	(−42, −54, 6)
	MOG_L	47	11.281	(−39, −87, 12)
	IFG_L	38	18.582	(−51, 30, 6)

*SMG_R, right supramarginal; MTG_L, middle temporal gyrus; MOG_L, middle occipital gyrus; IFG_L, left inferior frontal gyrus.*

### Correlation With Behavioral Function

**FIGURE 6:**
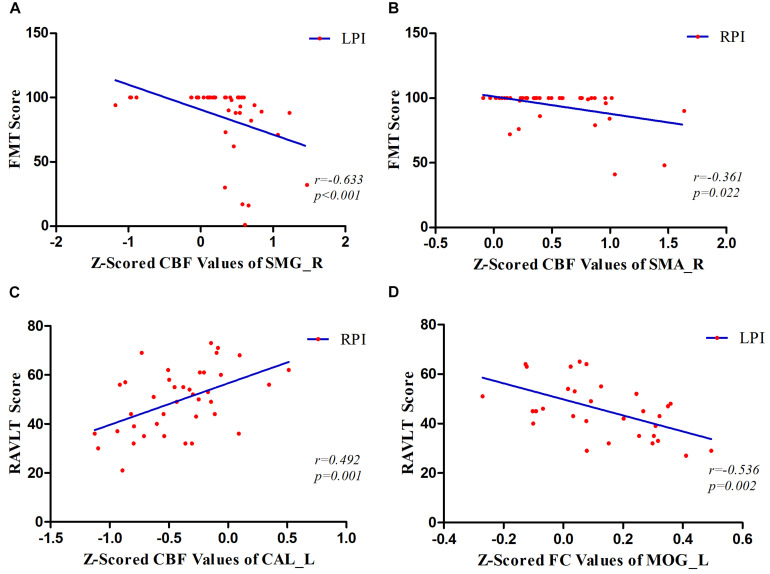
Correlations between *z*-scored CBF values, FC values, and behavioral scores over time. **(A)** The patients in the LPI group showed a significantly negative correlation between dynamic changes of *z*-scored CBF values in the SMG_R and FMT scores over time. **(B)** In the RPI group, the results showed a negative correlation between the longitudinal *z*-scored CBF values of SMA_R and FMT scores. **(C)** In the RPI group, the results showed a positive correlation between longitudinal changes of *z*-scored CBF values in the CAL_L and RAVLT scores. **(D)** In the LPI group, the longitudinal *z*-scored FC values between SMG_R as seed and MOG_L were negatively associated with RAVLT scores. SMG_R, right supramarginal gyrus; SMA_R, right supplementary motor area; CAL_L, left calcarine sulcus; MOG_L, left middle occipital gyrus; FMT, Fugl–Meyer Test; RAVLT, Ray Auditory Verbal Learning Test; LPI and RPI, left and right pontine infarction groups.

The correlations between CBF values in brain regions with longitudinal CBF differences and functional improvement are shown in Figure 6. We found a significantly negative correlation between dynamic CBF changes in the SMG_R and improvement in motor scores in the LPI group (*r* = −0.633, *P* < 0.001). In the RPI group, the *z*-scored CBF values of the SMA_R showed a significantly negative correlation with motor recovery (*r* = −0.361, *P* = 0.022), and the *z*-scored CBF values of CAL_L showed a significantly positive correlation with verbal memory functional improvement (*r* = 0.492, *P* = 0.001). In addition, correlations between the FC values and behavioral scores over time were also investigated (Figure 6). The *z*-scored FC values between the SMG_R and MOG_L were correlated with verbal memory RAVLT scores (*r* = 0.536, *P* = 0.002).

## Discussion

In this study, by combining pcASL and rs-fMRI, we investigated the longitudinal changes in CBF and FC during the 6 month follow-up period. These findings suggested that abnormal perfusion first appeared in the acute period and that the LPI and RPI groups at the baseline stage showed significantly lower *z*-scored CBF values in the bilateral cerebellum and abnormal perfusion in some supratentorial brain regions, as compared with the NC group. The longitudinal study showed the significantly altered CBF in the SMG_R for the LPI group and in the CAL_L, MOG_L, and SMA_R in the RPI group. We further demonstrated that the abnormal perfusion was concomitant with dynamic FC changes during functional recovery. Longitudinal FC changes were found in the MTG_L, MOG_L, and IFG_L when the SMG_R was used as the seed in the LPI group. In addition, these longitudinal changes in the *z*-scored CBF and FC values were associated with behavioral recovery, including motor and cognitive functions, over time. These results may promote our understanding of the neural pathophysiological underlying recovery from PI.

### Cross-Sectional CBF Differences Between PI and NC Subjects

Some cross-sectional studies have investigated abnormal CBF in chronic cortical or subcortical stroke patients (Miao et al., 2018; Obayashi, 2019). A location-dependent study is essential for understanding particular patterns of brain impairment and reorganization after stroke since the location of the lesion plays a vital role in patients’ functional outcomes. Our previous studies on CBF changes in chronic PI showed that significantly lower perfusion in the bilateral cerebellum and abnormal perfusion in the ipsilesional cerebellum were associated with motor scores (Wang et al., 2019). In this study, patients with acute LPI showed significantly lower *z*-scored CBF values in the bialteral cerebellum (left lobule VIII, Crus I, and right lobule VIIB) and ITG_R, MTG_R, IFG_L, and INS_L, and higher *z*-scored CBF values in the PCUM_R and SPG_L. On the other hand, patients with RPI showed significantly lower *z*-scored CBF values in the bilateral cerebellum (left lobule VIII, right lobule VI, and Crus II) and higher *z*-scored CBF values in the SFG_R and PreCG_R at the baseline stage. Coupled with these results, we argued that hypoperfusion of the bilateral cerebellum first appeared at the acute stage and maintained low-level perfusion to the chronic phase. Previous studies have reported that the cerebellum is involved in various motor and cognitive functions, cerebellar anterior lobe lesions may cause the cerebellar motor syndrome, and cerebellar posterior lobe lesions may produce the cerebellar cognitive affective syndrome (Schmahmann, 2004). Therefore, continuous hypoperfusion may be one of the factors in impaired motor and cognitive function after PI. We also found different patterns of CBF changes in some supratentorial brain regions in the LPI and RPI groups. Previous observations have shown that abnormal perfusion of the frontal parietal and temporal areas commonly occurs after unilateral brainstem stroke. Moreover, some studies suggested that factors such as lesion size, number, and location may affect the occurrence of supratentorial hypoperfusion, in which upper PI is more likely to cause ipsilateral supratentorial hypoperfusion, while contralateral supratentorial hypoperfusion is common in the middle pontine to medulla infarctions (Fazekas et al., 1993; Miyazawa et al., 1999; Matsui et al., 2007; D’Aes and Marien, 2015). This may partly explain the inconsistence of supratentorial perfusion patterns in the LPI and RPI groups.

### Longitudinal Alterations of CBF in the LPI and RPI Groups

A longitudinal study is an effective way to explore the neurobiological underpinnings of clinical recovery. In our longitudinal investigation, the LPI group showed CBF changes in the SMG_R from baseline to 6 months after infarction and significantly lower *z*-scored CBF values at the 6 months. The SMG serves as the core region of attentiveness, motor function, and spatial perception (Ciaramelli et al., 2008; Golay et al., 2008). Area-specific cytoarchitecture patterns were investigated in a previous study, which demonstrated that the SMA and pre-SMA were strongly co-activated with the SMG (Ruan et al., 2018). Moreover, a study of chronic stroke patients with upper limb hemiparesis showed better recovery of motor function when the cortical thickness of the SMG was thinner (Ueda et al., 2019). These studies further validated our findings that the CBF changes in the SMG_R correlated with motor recovery after stroke and that the abnormal perfusion of the SMG_R offers a novel approach to explore the neural mechanisms of behavioral recovery. The RPI group showed significant CBF changes in the ipsilateral sensorimotor cortex and contralateral visual cortex, and these brain regions displayed different trends over time. In addition, the dynamic CBF changes in the SMA_R and gradually increased perfusion in the CAL_L were associated with functional recovery in motor and cognitive memory. Some previous studies have demonstrated that memory and attention interact. Rotshtein et al. (2011) suggested that the FC from the thalamus to the visual cortex is associated with working memory performance. Soto and Humphreys (2007) identified increased neural responses in early visual cortices when searching for reappearance of an item from working memory. Thus, the higher *z*-scored CBF values in the CAL_L may underlie memory recovery over time. Previous studies also have suggested a central role of SMA in the motor network, and early structural and functional changes of SMA are an important predictor of long-term motor outcome (Hannanu et al., 2017; Du et al., 2018). Thus, abnormal perfusion of SMA_R may indicate a neural mechanism of motor recovery after RPI.

At present, the detailed mechanisms underlying CBF changes in local brain regions remain unclear. One possible explanation is the destruction and reorganization of neurovascular coupling. Numerous studies have demonstrated that neural activity is tightly associated with CBF, and the close temporal and spatial relationship between neural activity and CBF is termed “neurovascular coupling.” However, the balance between neural activity and CBF is disrupted after stroke, resulting in hemodynamic abnormalities (Blicher et al., 2012; Bohm et al., 2020). Another possible reason is the disruption of white matter fiber integrity after PI, which may cause progressive anterograde and retrograde degeneration of fiber bundles between original infarction site and the remote regions (Liang et al., 2008). These processes could eventually lead to abnormal CBF patterns in remote regions.

### Longitudinal FC Changes in the LPI and RPI Groups

A number of studies have proposed that abnormal perfusion in local brain regions may be a potential cause of local brain structural and functional changes (Yu et al., 2017; Zheng et al., 2019). In this study, we also investigated progressive FC changes during the follow-up period, by taking brain regions with longitudinal CBF changes as seed-ROIs. We found longitudinal FC changes between seeded SMG_R and some regions in the LPI group, including MTG_L, MOG_L, and IFG_L. These FC changes showed a similar trend, gradually decreasing to a minimum at 3 months and then increasing thereafter. Several previous studies have investigated the neural bases of verbal memory and have demonstrated that the left-lateralized frontoparietal network, superior temporal and occipital lobe cortices, and cerebellum are involved in the verbal memory process and that the left SMG is one of the key regions (Emch et al., 2019; Koshy et al., 2019). Impaired verbal memory after disease may lead to functional and structural changes in the homologous brain regions to maintain processing. In our study, the dynamic changes in FC between the SMR_R and MOG_L were associated with RAVLT scores over time in the LPI group. This phenomenon may be a compensatory mechanism aimed at maintaining and promoting the recovery of memory function.

This study had some limitations. First, due to the strict inclusion criteria, the sample size was relatively small, and some studies have suggested that the clinical manifestation and prognosis vary according to the level of PI (Jang, 2012; Huang et al., 2019). In addition, the small sample size also limited the statistical power. Therefore, in further studies, it is necessary to recruit patients with the different level pontine lesions to investigate the mechanisms of behavioral recovery. Second, the recovery of clinical function is a long-term process, and a relatively short follow-up period may be insufficient to observe the exact trends of clinical recovery. We therefore plan to observe these patients in the longer term. Third, in this study, we explored the abnormal perfusion as well as FC in PI during the 6-month follow-up by using ASL and BOLD-fMRI. However, we did not further calculate FC values based on brain regions with abnormal spontaneous BOLD signal oscillation as seeds. Further, we will extend this study through further analysis of BOLD signal changes to comprehensively assess spontaneous brain activity after PI.

In conclusion, we investigated longitudinal alterations of regional CBF and FC in patients with PI. Significantly abnormal perfusion first appeared at the acute stage after infarction. Longitudinal results demonstrated that patients with LPI showed abnormal CBF changes in the SMG_R, while RPI groups showed significant CBF changes in the CAL_L, MOG_L, and SMA_R. Moreover, dynamic CBF changes were accompanied by FC changes during the follow-up period. Correlation analysis demonstrated that longitudinal changes in CBF and FC were significantly correlated with motor or cognitive scores over time. These longitudinal alterations enable us to better understand the neurobiological mechanisms of behavioral recovery after PI.

## Data Availability Statement

The raw data supporting the conclusions of this article will be made available by the authors, without undue reservation.

## Ethics Statement

The studies involving human participants were reviewed and approved by the First Affiliated Hospital of Zhengzhou University and Tianjin Medial University General Hospital. The patients/participants provided their written informed consent to participate in this study.

## Author Contributions

YWe, CW, JL, and JC conceived and designed the experiments. YWe, JL, CW, PM, LW, and YWa performed the MRI scans and behavioral assessment. YWe, KW, and JC wrote the manuscript. All authors contributed to the article and approved the submitted version.

## Conflict of Interest

KW was employed by the company GE Healthcare. The remaining authors declare that the research was conducted in the absence of any commercial or financial relationships that could be construed as a potential conflict of interest.

## Footnotes

1 www.nitrc.org/projects/mricron

2 http://www.Fil.ion.ucl.ac.uk/spm/software/spm

3 http://rfmri.org/dpabi

4 restfmri.net/forum/
