# Supplementary figures and images for: Disrupted Regional Cerebral Blood Flow and Functional Connectivity in Pontine Infarction: A Longitudinal MRI Study
Source: Front Aging Neurosci. 2020 Nov 19;12:577899. doi: 10.3389/fnagi.2020.577899 (PMC7710811; doi:10.3389/fnagi.2020.577899)

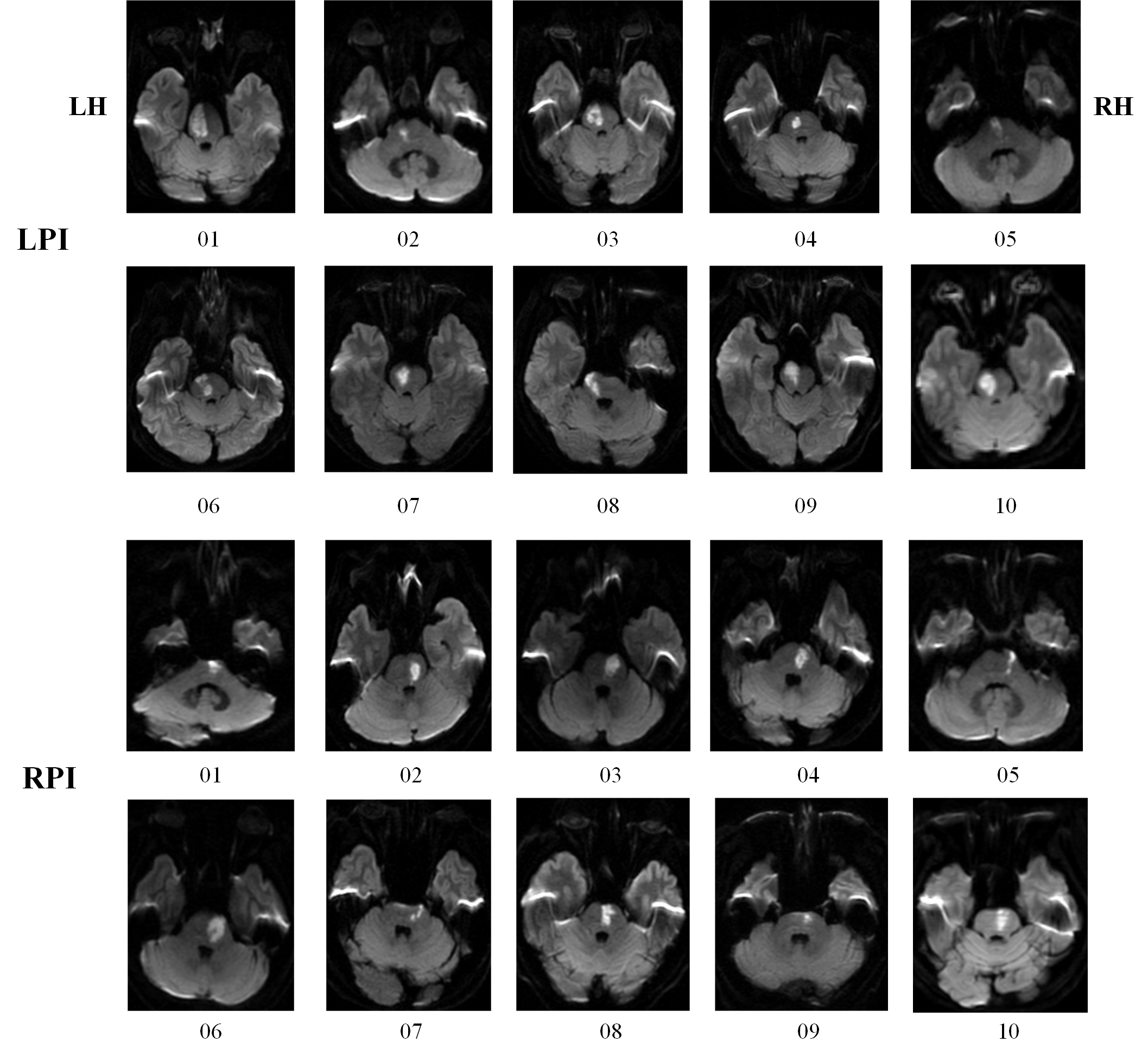

Supplement: Supplementary file 1 [file Image_1.TIF]
