# Supplementary material for: Disrupted Regional Cerebral Blood Flow and Functional Connectivity in Pontine Infarction: A Longitudinal MRI Study
Source: Front Aging Neurosci. 2020 Nov 19;12:577899. doi: 10.3389/fnagi.2020.577899 (PMC7710811; doi:10.3389/fnagi.2020.577899)
Supplement: Supplementary file 2 [file Table_1.DOCX]

Table S1 Demographic and Clinical Data in the LPI and RPI groups

| Patient No. | Age | | Sex | EducationYears | Main Symptoms | | Infarction Location | |
| --- | --- | --- | --- | --- | --- | --- | --- | --- |
| *LPI group*  01  02  03  04  05  06  07  08  09  10  *RPI group*  01  02  03  04  05  06  07  08  09  10 | | 69  57  59  56  61  63  57  62  63  54  51  47  62  54  44  53  67  56  46  64 | F  F  F  M  M  M  M  M  M  M  F  F  F  M  F  F  M  M  M  F | 6  12  6  6  15  9  15  12  9  12  9  9  9  9  12  12  9  9  12  6 | | Right hemiparesis  Right hemiparesis, dizziness  Right hemiparesis, dysarthria  Right hemiparesis, dizziness, dysarthria  Right hemiparesis, dizziness, dysarthria  Right hemiparesis, dysarthria  Right hemiparesis, dysarthria  Right hemiparesis, dysarthria  Right hemiparesis  Right hemiparesis, dizziness  Left hemiparesis, dizziness  Left hemiparesis, dizziness  Left hemiparesis  Left hemiparesis, dysarthria  Left hemiparesis, dizziness  Left hemiparesis, dysarthria  Left hemiparesis, dysarthria  Left hemiparesis, dysarthria  Left hemiparesis, dysarthria  Left hemiparesis | | Left ventromedial upper pontine  Left ventrolateral lower pontine  Left ventromedial medial pontine  Left ventromedial lower pontine  Left ventrolateral lower pontine  Left ventrolateral middle pontine  Left ventromedial middle pontine  Left ventromedial lower pontine  Left ventrolateral upper pontine  Left ventromedial lower pontine  Right ventrolateral lower pontine  Right ventrolateral lower pontine  Right ventromedial lower pontine  Right ventromedial lower pontine  Right ventrolateral lower pontine  Right ventromedial lower pontine  Right ventrolateral lower pontine  Right ventrolateral lower pontine  Right ventrolateral lower pontine  Right ventromedial upper pontine |
